# Comparative Study on Mixing Behavior of Binary Mixtures of Cocoa Butter/Tristearin (CB/TS) and Cocoa Butter/Coconut Oil (CB/CO)

**DOI:** 10.3390/foods9030327

**Published:** 2020-03-11

**Authors:** Bhagyashri L. Joshi, Birgitta I. Zielbauer, Thomas A. Vilgis

**Affiliations:** Max-Planck-Institute for Polymer Research, Ackermannweg 10, 55128 Mainz, Germany; birgitta.zielbauer@gmx.de (B.I.Z.); vilgis@mpip-mainz.mpg.de (T.A.V.)

**Keywords:** cocoa butter, coconut oil, tristearin, mixing behavior, morphology, phase diagram, DSC, PLM

## Abstract

The comparative study between the mixing behavior of two binary mixtures of cocoa butter (CB)/tristearin (TS) and cocoa butter (CB)/coconut oil (CO) was investigated by using differential scanning calorimetry (DSC). The DSC profile for CB/TS blends resulted in a monotectic temperature–concentration (T–X) phase diagram, whereas a phase diagram of eutectic type was observed for CB/CO blends at 65 wt % of CO and 35 wt % CB; this suggests that the eutectic crystal can be formed when the saturated fat (blue = CO) is smaller in size compared to monounsaturated fat (orange = CB), whereas, for similar and larger size (red = TS) to CB, phase separation under crystallization is likely to occur (as shown in the graphical abstract). In order to understand the interaction between the binary systems, the profile of the phase diagram was fitted with Bragg–Williams approximation for estimation of the nonideality mixing parameter. Moreover, the morphology of the two different systems by polarized light microscopy (PLM) also depicted the variations in phase behavior by showing a significant change in CB morphology from spherulitic, grainy to granular and needlelike after the addition of TS and CO, respectively. Our findings emphasize the fundamental understanding of the interaction of bulk fat/fat and fat/oil system.

## 1. Introduction

The mixing of fats and oils is widely used in food, pharmaceutical and cosmetic applications [1,2,3]. Likewise, in some applications, the lipid system is one of the main constituents, which defines the ultimate product quality and storage life [4,5]; therefore, the interaction between different fats and oils in mixtures of various compositions is important to design the desired product having specified physicochemical properties. Apart from these application aspects, studies of fat and oil blends offer deep insight into the fundamental aspects of the crystallization of molecules with molecularly controlled complexity.

Fats and oils mainly consist of mixtures of triacylglycerols (TAGs), having three fatty acids attached to the glycerol backbone, hence, their physicochemical properties depend on the sn–position of these fatty acids to the glycerol backbone and their degree of saturation [6]. This specific structure of the TAGs allows the direct observation of crystallization phenomena due to the slow time scales expressed in significant slow crystallization rates, which are controlled by the length of the fatty acids and the saturation degree. Many relevant physical quantities can thus be directly observed.

In the case of TAGs, typically three (α, β′ and β) different polymorphs form upon cooling [7]; therefore, depending on the packing density of the TAGs (loosely packed or densely packed) the physicochemical properties of fat crystals get affected [8]. It is necessary, therefore, to understand the interactions inside binary systems in liquid and solid-states for developing various applications. Although the mixing behavior of various pure TAGs has been explored very well [9,10] and their phase diagram has also been studied in detail, there are only a few studies that explain the thermodynamic analysis of the mixing behavior in bulk systems [11]. Therefore, in this study, we have considered two sets of blends cocoa butter (CB)/tristearin (TS) and cocoa butter (CB)/coconut oil (CO) to understand the interaction between these mixtures on a broad concentration spectrum.

CB consists of mainly monounsaturated symmetrical TAGs, namely POP (1,3-dipalmitoyl-2-oleoyl glycerol), POS (1,3-palmitoyl-stearoyl-2-oleoyl glycerol) and SOS (1,3-distearoyl-2-oleoyl glycerol); where P = palmitic acid, O = oleic acid and S = stearic acid (approximately 17% of POP, 37% of POS and 27% of SOS) [12]. The remaining part consists of mixtures of monounsaturated and polyunsaturated TAGs, as well as traces of polyphenols and free fatty acids. CB crystallizes into six (γ, α, β_2_′, β_1_′, β_2_ and β_1_) different polymorphs in which melting temperature and their stability increases from γ to β_1_ [13].

TS is a monosaturated TAG composed of three stearic acids (SSS) with 18 carbon atoms each, which crystallizes into three polymorphs namely, α (less stable), β′ (metastable) and β (highly stable) with melting temperatures of 54 °C, 65 °C and 72.5 °C, respectively [14]. Previous studies were only carried out on the addition of a low concentration of TS to CB. For instance, the study on the effect of 1 wt % and 5 wt % of TS on the crystallization of CB showed the increase in onset temperature of the mixture and the polymorphic transition from β′ to β was also delayed [15]. However, systematic investigations on higher percentages have not yet been explored; hence, in our present study, the selection of such a combination was used. Interestingly, TS is also present in CB as a minor component [12], and hence, it was intriguing to observe the possible changes that would occur after the blending of TS in a higher percentage with CB.

It has been shown that CO can be used as a cocoa butter substitute [16,17] as it consists of a high amount of lauric acid (~40%) followed by myristic and palmitic acid [18]. There are a wide range of research studies that have been done previously on mixing behavior and crystallization of CB with lauric fats [19,20,21,22]. However, the effect of isothermal and dynamic crystallization processes on physicochemical properties and morphological changes of a broad range of CB/CO blends has not been explored yet. We believe that following this approach will help to determine the exact eutectic point of CB/CO blends.

**Figure 1:**
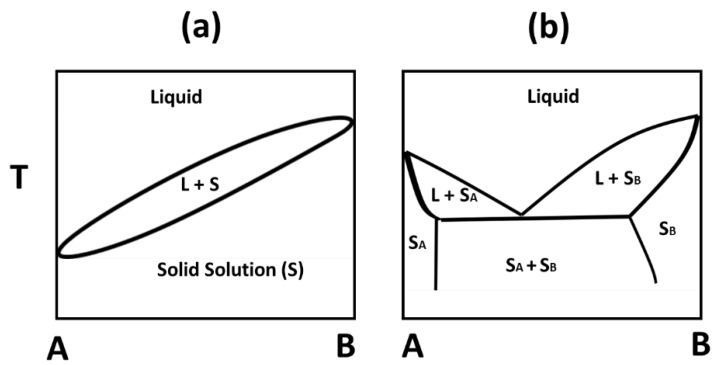
Model schematic of two types of phase diagram in binary mixtures of triacylglycerols (TAGs); (**a**) monotectic, continuous solid solution, (**b**) eutectic phase behavior (redrawn from [23] with copyright permission).

The mixing ratios of a binary system of fat–fat and fat–oil blends determine their solid–liquid phase diagram. The solid–liquid phase diagram helps to understand the differences in the thermal properties of blends when mixed together in different ratios. There are typically four different phase diagrams identified by Timms [23]—(1) monotectic continuous solid solutions, (2) eutectic systems, (3) monotectic partial solid solutions, and (4) peritectic systems. Interestingly, in our study, CB/TS and CB/CO blends showed monotectic and eutectic mixing behavior, respectively. The model schematic is shown in Figure 1 for typical monotectic and eutectic phase diagrams.

However, in the case of pure saturated fat, one may have to consider either melt-mediated or solid–liquid–solid (S–L–S) transition or solid–solid (S–S) transition while constructing the solid–liquid phase diagram. Melt-mediated, also known as S–L–S transition, can be explained by the transformation of one polymorph into another via melting. During heating, one polymorph melts and recrystallizes into another polymorph. On the contrary, S–S transition is the transformation of one polymorph into another form in solid state [24]. Understanding such transformations could help to design the technological processes during fat crystallization in food applications.

Altogether we analyzed the thermal properties of different blends of CB/TS and CB/CO mixtures by using isothermal and dynamic crystallization methods. The changes in thermal properties and morphology of these blends were measured by using differential scanning calorimetry (DSC) and polarized light microscopy (PLM), respectively. From the DSC results, the phase diagram was constructed. The thermodynamic analysis of these phase diagrams helped to understand the interaction between CB/TS and CB/CO in a solid and liquid state. Therefore, our study sheds a light on the applied point of view while understanding the basics of these interactions. Ultimately, it will help in future for comprehending the correlation of differences in mouthfeel, based on usage of different concentrations of the mixtures of these oil and fat blends.

## 2. Materials and Methods

### 2.1. Materials and Blends Preparation

To study thermal properties and morphological changes in the mixtures, CB (Carl Roth GmbH + Co. KG, Karlsruhe, Germany), TS and CO (Sigma–Aldrich GmbH, Steinheim, Germany) were used as a model system. The blends were prepared at ratios from 100 *w*/*w* % to 0 *w*/*w* % in steps of 10 wt % for each experiment. For the preparation of samples, CB and another fat were melted individually and then added together; this mixture was further heated for another 30 min while stirring. Later, the mixture was transferred into a preheated sample holder (aluminum crucible and microscopic glass slide for DSC and PLM, respectively). Afterward, the sample was heated again for 15 min in the oven to eliminate the crystallization due to temperature difference, while transferring the sample. The sample was then stored for 24 h at 22 °C in a constant climate chamber (Binder KMF115) with 0% relative humidity. The analysis of these samples was carried out by using DSC and PLM for thermal analysis and morphological studies, respectively.

### 2.2. Compositional Analysis of CB and CO

The fatty acid analysis of CB and CO was determined by GC-FID commercially (Company A, name not mentioned due to confidentiality agreement) and TAG composition of CB was resolved by HPLC analysis commercially (Eurofins Analytik GmbH, Hamburg, Germany).

### 2.3. Thermal Analysis by Differential Scanning Calorimetry (DSC)

DSC was used for the thermal analysis of the mixtures. The blends were prepared as explained in Section 2.1 and then stored in the aluminum crucible (~20 mg sample) for 24 h at 22 °C prior to DSC (Mettler Toledo DSC3+/700/453) measurements. An empty 100 µL aluminum pan was considered as a reference cell. Liquid nitrogen was used for cooling with a rate of 30 mL/minute. The experiment was performed in four segments in order to study the thermal properties of fat/oil blends crystallized via isothermal and dynamic processes. The sample, which was crystallized by the isothermal process at 22 °C, was heated first to 90 °C in order to study the crystal formation after 24 h and kept for 20 min to erase all crystal memory. Later, the sample was cooled down to −50° C and immediately heated to 90 °C in the next segment. In our study, we have considered the peak temperature as the melting/crystallization temperature of the system, and the onset and offset point denotes the start and end of the thermal process. For a better understanding of the DSC profile, the heat flow parameter was increased in steps of 0.2 W/g in each sample measurement while plotting in Section 3.2.1. All thermograms were plotted with respect to weight percent (X_CB_) of CB in the blends. The analysis was performed in triplicate, and the evaluation of the graphs was carried out in STARe software.

### 2.4. Thermodynamic Analysis of Pseudophase Diagrams

Based on DSC results, we generated the pseudophase diagram for CB/TS and CB/CO blends. The reason to term these binary phase diagrams as ‘pseudo’ is that each component of the ‘blended’ CB and CO consists already of broader mixtures of TAGs, which makes them a bulk system and not a pure single-component system. However, in our case and according to the data obtained, we assume CB and CO as a one constituent system, and therefore our diagram reflects the binary phase diagram.

The phase behavior of binary systems has been explained in detail for fatty acid mixture and phospholipids by using ideal mixing and nonideality of mixing [25,26,27]. For ideal mixing behavior, the Hildebrand equation is used to simulate the liquidus line derived from the phase diagram [26,28]. According to this model, the liquidus line of a binary mixture of components A and B being completely immiscible in the solid phase, is given by either Equation (1) or (2), depending on their composition range [28,29].

(1) $$\ln x_{A}=-\frac{∆H_{A}}{R}\left(\frac{1}{T}-\frac{1}{T_{A}}\right)\;$$

(2) $$\ln x_{B}=-\frac{∆H_{B}}{R}\left(\frac{1}{T}-\frac{1}{T_{B}}\right)\;$$

where *R* is the gas constant, $x_{A}$, $∆H_{A}$, $T_{A}$ represent, mole fraction, molar latent heat of fusion and melting point of A, respectively, similarly, $x_{B}$, $∆H_{B}$, $T_{B}$ for component B.

On the other hand, the nonideality of mixing is described by the Bragg–Williams approximation. This equation is based on the nonideality parameter χ, which explains the interaction between two components in liquid and solid-state. χ is the energy difference between the pair (A–B) and the average of (A–A) and (B–B) pairs and is described as

(3) $$\chi \;=z\;\left(u_{AB}-\frac{\left(u_{AA}+u_{BB}\right)}{2}\right)\;$$

where z is the coordination number, i.e., the number of nearest-neighbor molecules of the individual molecules and $u_{AB}$, $u_{AA}$ and $u_{BB}$ are the binary interaction energy of the pair A–B and pair A–A and B–B, which describes the interaction of either component in liquid or a solid phase. In principle, these energies reflect the point-like pseudo interaction between monomers in polymer mixing theory [30], however, in case of mixed TAGs, the monomers (fatty acids) are not of similar origin hence, these interactions could be either between the methyl end group, aliphatic carbon–carbon chain length or from esterified glycerol part of different TAGs. Therefore, these fatty acids cannot be considered as point-like in the lattice model for understanding the interaction. Thus, $u_{AB}$, $u_{AA}$ and $u_{BB}$ would be assumed as the combination of interactions between three different parts of TAG, as explained above.

For ideal mixing, the difference is zero, therefore, χ is zero. Positive χ indicates the clustering of similar molecules, which beyond some critical value leads to phase separation into phases of different composition. Negative χ denotes a tendency for order [26]. The two equations which are shown below represents the Bragg–Williams approximation of nonideality;

(4) $$\ln x_{A}+\;\chi {\left(1-X_{A}\right)}^{2}/RT=-\frac{∆H_{A}}{R}\left(\frac{1}{T}-\frac{1}{T_{A}}\right)\;$$

(5) $$\ln x_{B}+\;\chi {\left(1-X_{B}\right)}^{2}/RT=-\frac{∆H_{B}}{R}\left(\frac{1}{T}-\frac{1}{T_{B}}\right)$$

For monotectic behavior, either of the two equations is simulated to give the liquidus line, whereas, for the eutectic mixture, for composition range between $x_{E}\;\leq x_{A}\leq 1$ Equation (4) is used and for $0\;\leq x_{A}\leq x_{E}$ Equation (5) is used.

In our study, we have considered A and B components as TS and CB in CB/TS blends, respectively, and CB as A and CO as B in CB/CO blends. For calculation of the mole fraction of CB and CO, the average molecular weight was calculated based on the TAG profile of both lipids. The TAG profile for CB was evaluated as given in Section 2.2, and for CO the TAG profile was taken from the study of Toro–Vazquez group study on physicochemical properties of trans-free and partially hydrogenated soybean oil [31]. The calculated average molecular weight of CB, CO and TS were 822 g/mol, 627 g/mol and 890 g/mol, respectively. For fitting these equations to the phase diagram derived from DSC, OriginPro 9.1 software (OriginLab Corporation, Northampton, MA, USA) was used.

### 2.5. Morphological Studies by Polarized Light Microscopy (PLM)

The morphology of the samples was studied by PLM (Zeiss Scope, A1 Pol). A 10 µL of sample was pipetted to a preheated microscope slide, and carefully, the coverslip was placed on the top of the molten sample to avoid air bubble formation. These slides were stored at 22 °C for 24 h prior to PLM. The pictures were captured after 24 h for each sample, and the result of dynamic crystallization was studied by using a temperature profile similar to the one used in DSC analysis. The heating and cooling were attained by a Peltier plate setup (Linkam, model PE120). The sample was heated to 90 °C with a 2 K/minute rate and kept isothermally for 60 min to erase the crystal memory. As the heating systems are different in DSC and PLM, longer isothermal heating time at 90 °C was used as compared to DSC. Afterward, the sample was cooled down to 10 °C with a 2 K/minute rate, and at this temperature, the pictures were captured by using objective 20×. All measurements were carried out in duplicate.

## 3. Results and Discussions

### 3.1. Compositional Analysis of CB and CO

The fatty acid (FA) analysis of CB and CO is shown in Table 1. The results depict that CB consists of long-chain saturated FA ranging from (C18:0)–(C24:0). The concentration of stearic acid (C18:0), arachidic acid (C20:0), behenic acid (C22:0) and lignoceric acid (C24:0) are significantly high as compared to other studies [32,33]. As a result, this kind of CB could melt at a higher temperature. Thus, while preparing the samples, the temperature used was 90 °C, to ensure complete melting. The concentration of palmitic acid (25.32 wt %), stearic acid (36.74 wt %) and oleic acid (32.48 wt %) in CB is highest with respect to other FAs present in CB, which is in agreement with other studies [34,35,36]. In contrast with CB, the concentration of (C18:0)–(C24:0) was very low in CO, and it mainly consisted of small and medium-chain FA ranging from (C8:0)–(C16:0), in which lauric acid (42.78%) and myristic acid (17.64%) were present in higher concentration. Although CO was found to contain a high amount of saturated fatty acids, oleic acid (9.23%) and linoleic acid (2.99%) were also detected in a significant amount. This mixture of short, medium and long-chain fatty acids and their sn–position explains the different physical, thermal and mechanical properties of fats [6].

The TAG composition of CB was determined by HPLC and is shown in Table 2. However, the TAG composition did not show the presence of arachidic acid (C20:0), behenic acid (C22:0) and lignoceric acid (C24:0) in terms of TAGs. Unfortunately, we could only succeed to separate ~95% of TAGs; therefore, it was difficult to conclude whether these long-chain fatty acids were present in TAG, diacylglycerol (DAG), monoacylglycerol (MAG) or in terms of free fatty acid. Thus, the mixtures of different TAG, DAG, MA and FA in CB form were a complicated system by itself.

Therefore, the thermal properties of fats and oils mainly depend on the composition of the individual fat and wt % in the mixture. For instance, blends of CB/canola oil and CB/soybean oil show different characteristics, as canola oil consists of mainly OOO, LOO and OLnO, whereas in soybean oil LLL, LLO and LOO are present (where O = oleic acid; L = linoleic acid; Ln = linolenic acid) [37].

### 3.2. Characterization of Thermal Properties by Using DSC

#### 3.2.1. CB/TS Blends Characterization

**Figure 2:**
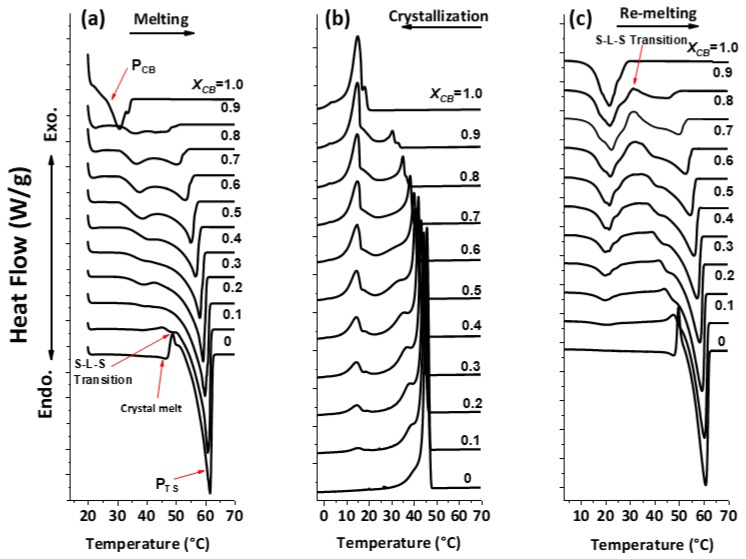
Differential scanning calorimetry (DSC) analysis of cocoa butter (CB)/tristearin (TS) (CB/TS) blends. (**a**) Melting process after crystallization for 24 h at 22 °C. The arrows indicate the peak of CB (PCB), the peak representing TS (PTS) and α polymorph melting. (**b**) Dynamic crystallization from 90 °C to −50 °C. (**c**) Remelting of crystals formed in segment (**b**). Arrow indicates the S–L–S (Solid–Liquid–Solid) transition.

Figure 2 shows DSC thermograms for three different segments of CB/TS mixtures. After storage of the sample at 22 °C for 24 h, the melting thermogram (Figure 2a) was performed for studying the effect on the melting behavior of blends after isothermal crystallization. CB/TS showed a linear trend with respect to change in concentration of fats. In the case of pure CB, two endothermic peaks occurred during the melting process, which can be ascribed to the melting of two polymorphs, form IV (30.85 °C) and form V (33.6 °C) [13]. Similarly, in the case of pure TS, one endothermic peak at 46.8 °C (indicated by α melt arrow) and another at 61.7 °C (P_TS_) were observed. However, according to a study by Windbergs’ group [14], onset temperatures of 54 °C and 63 °C represent α and β′ polymorphic forms. The difference in the melting temperatures between our work and Windbergs’ study might correspond to the difference in sample preparation technique.

For blends, after the addition of TS to pure CB, the peak shifted towards a higher temperature, and the peak area increased accordingly as the concentration of TS increased. Another observation, in the pure TS curve, an exothermic peak was observed during melting (indicated with a red arrow in Figure 2a). This phenomenon occurs due to S-L-S or melt-mediated transition in TS. Firstly, one crystal form was melted at 46.8 °C and recrystallized into another crystal form at 48 °C, which was melted immediately. Therefore, an observable S–L–S transition instead of S–S transition occurs.

Similarly, with DSC and X-ray diffraction, the study of Lavigne and group proved the hypothesis of α to β phase transition in TS was S–L–S transition type, as the β phase occurred from a melt of α and not directly from solid α [38]. The S–S transformation from vertical α form to β’ could be due to the collapse of hydrocarbon chains or from bending of each molecule in the glycerol region [39]; however, the reason for S–L–S transformation is still yet to understand.

The next segment (in Figure 2b) was designed to observe the effect of dynamic crystallization on the individual fats and their respective blends. The controlled cooling process was achieved by this segment led to the formation of different crystal forms from the isothermal crystallization for 24 h. The crystallization exotherms for all samples are shown in Figure 2b. Pure CB (X_CB_ = 1) crystallized according to the composition of TAGs present in the CB. The cocrystallization of the high melting fraction (HMF), a medium melting fraction (MMF) and a low melting fraction (LMF) took place. Likewise, pure TS crystallized into one crystal form, having a higher melting point and showing a sharp peak at 45 °C, along with the shoulder peak of another polymorph. The blends produced two exothermic peaks, in which P_TS_ represented TS, and P_CB_ indicated CB. Furthermore, P_TS_ shifted towards lower temperature after the addition of CB into it, and the area of peak decreased accordingly. Whereas, for P_CB_, only the peak area decreased after the addition of TS, but not specific change in the crystallization temperature of CB was observed.

For determination of the ∆H value in crystallization and melting segments of CB/TS blends, P_CB_ peaks were taken as reference peaks, as there was no specific peak shift. For the crystallization process, the range from 90 °C to 24 °C was considered for P_TS_, and below for CB, whereas, for the remelting process, first endothermic peak area for CB, exothermic peak for solid–liquid–solid transition and second endothermic peak for TS (refer to supplementary Figure S1).

**Figure 3:**
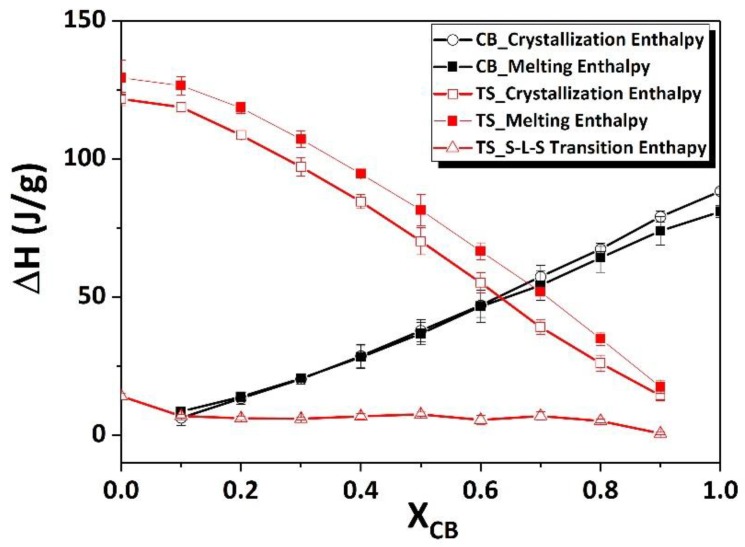
Enthalpy change of CB and TS during the crystallization and remelting process with respect to weight % of CB. black open square = crystallization, black filled square = remelting, red open square = crystallization, red filled square = remelting, open triangle = enthalpy change in S–L–S transition while remelting.

The remelting procedure was carried out to determine the melting profile of crystals that formed during the crystallization process (segment 2) with a heating rate of 2 K/minute. The melting endotherms detected by DSC are shown in Figure 2c. Similarly, as in segment 2, P_TS_ represents endotherm of TS and P_CB_ represents CB. In the case of pure CB, DSC detected one sharp peak along with shoulder peaks, which ascribed that mixture of different melting fractions, were melted (from LMF to HMF). In the case of TS, two melting fractions were formed, similarly, as of first melting (segment 1 in Figure 2a), only the peak temperature of higher melting fractions was slightly shifted from 61.4 °C to 60.97 °C, respectively. Hence, from the endotherms of all the blends and the melting temperature (MT) of TS decreased after the addition of CB, similar to segment 1. However, the exothermic peak (S–L–S transition) did not vanish completely, even after the addition of CB. For the evaluation of these endotherms, the temperature limit was selected as: below temperature 31.06 °C, the area was considered as CB, and above temperature 33.4 °C for TS. Figure 3 shows the crystallization, melting and S-L-S transition enthalpy change against X_CB_. Δ*H* for crystallization and melting exponentially decreased as CB increased, whereas, for CB, both processes showed a linear decrease in enthalpy as the concentration of TS increased. In the case of S-L-S transition from the remelting method, the enthalpy change was almost constant throughout the composition of TS. This result indicated that Δ*H* for S–L–S transition was independent of the composition (see supplementary data Section 2).

#### 3.2.2. DSC Results of CB/CO Mixtures

**Figure 4:**
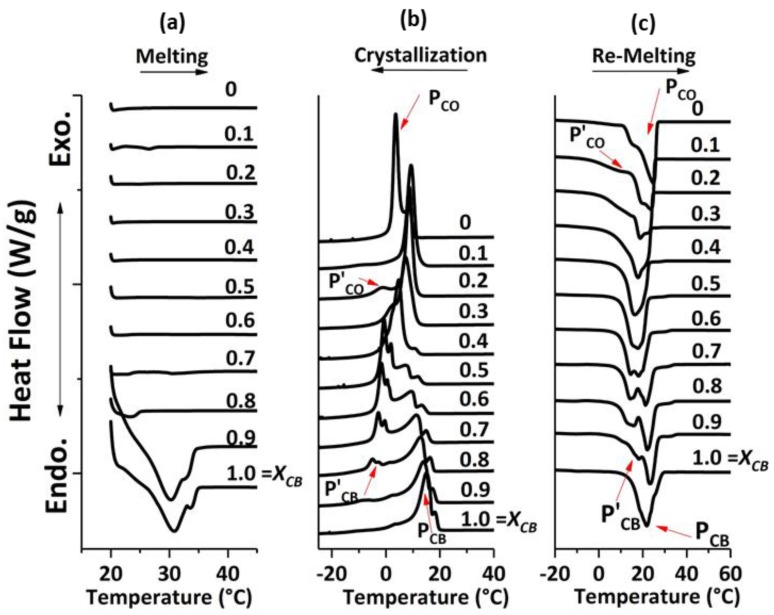
DSC analysis of CB/CO blends. (**a**) Melting process after crystallization for 24 h at 22 °C. (**b**) Dynamic crystallization from 90 °C to −50 °C. (**c**) Remelting of crystals formed in segment (**b**). The arrows indicate the peak of CB (P_CB_), the peak representing CO (P_CO_), shoulder peaks appearing after the addition of CO in CB (P’_CB_) and shoulder peaks appearing after the addition of CB in CO (P’_CO_).

Figure 4a–c show the DSC results of melting after 24 h at 22 °C, crystallization and remelting thermograms of CB/CO blends. In the first segment of melting, only the samples with X_CB_ of 1.0, 0.90 and 0.80 showed the melting from the crystals that were formed during 24 h of crystallization (Figure 4a). This behavior ascribed that CO was inhibiting the crystallization process of CB when added at more than 10 wt %. Similar results were obtained from NMR results studying the crystallization kinetics of CB and CO blends over a period of 24 h (refer to supplementary Figure S2). Likewise, the study [19] of crystallization behavior and kinetics of chocolate with lauric fat showed that at different temperature ranges, the solid fat content decreased after the addition of 10%, 20% and 30% of CO, palm kernel oil and fractionated palm kernel oil, respectively. This decrease in solid fat content can be explained by the TAG composition in both the fats. CO contains a mixture of mixed and short-chained saturated TAGs, whereas CB is a mixture of long-chain monounsaturated TAGs. Therefore, the packing ability is compromised between these fats together, and so eventually, the compatibility is reduced.

The results of crystallization are shown in Figure 4b. P_CB_ represents the main peak for CB in all mixtures; P’_CB_ indicates the shoulder peaks, which were appearing after the addition of CO in CB. Similarly, P_CO_ and P’_CO_ denote the main peak for CO and the shoulder peak in the blends, respectively.

As explained in Section 3.2.1, pure CB (X_CB_ = 1) crystallized in three fractions. Likewise, for pure CO (X_CB_ = 0), two crystallization exotherms were detected, in which one was a sharp peak at a lower temperature (2.5 °C), and the second peak overlapped with the same peak at a higher temperature (7.4 °C). This could be because of incomplete crystallization of different TAGs in CO with 2 K/minute cooling rate. If the rate of cooling decreases, there will be the possibility of complete crystallization of these short-chained mixed saturated TAGs (CLaLa, LaLaLa, LaLaM and CCLa), as they could get adequate time to arrange properly [40]. Further, up to the mixture of X_CB_ = 0.50, two different fats could be clearly identified, however, for X_CB_ = 0.40, the crystallization of all TAGs overlapped. Although it showed one main peak, there was still the existence of two shoulder peaks of HMF and LMF. For further blends, X_CB_ of 0.30, 0.20 and 0.10, the peaks are differentiated into P_CO_ for CO and P’_CO_ for shoulder peak, which appeared after the addition of CB.

The melting profile of these mixtures was then studied. Figure 4c shows the endotherm results of all the mixtures. Similar to crystallization, the shoulder peak developed after the addition of CO in CB, however, the identification of these peaks was only possible up to X_CB_ = 0.70. For 0.60 and 0.50, the overlapping of melting peaks started to occur. For a mixture of X_CB_ = 0.40, endotherm resulted in one main peak along with small shoulders. For X_CB_ of 0.20 and 0.10, P’_CO_ indicates the shoulder peak along with P_CO_ as a main peak in the mixtures. Hence from both crystallization and melting profile, the mixture at X_CB_ = 0.40 can be described as the eutectic mixture, however, in the case of crystallization, the peak for this concentration showed a broad profile instead of a sharp peak. Hence to determine the exact concentration profile for showing a pronounced eutectic mixture and so the eutectic point, we measured three more compositions, namely X_CB_ = 0.34, 0.35 and 0.36.

**Figure 5:**
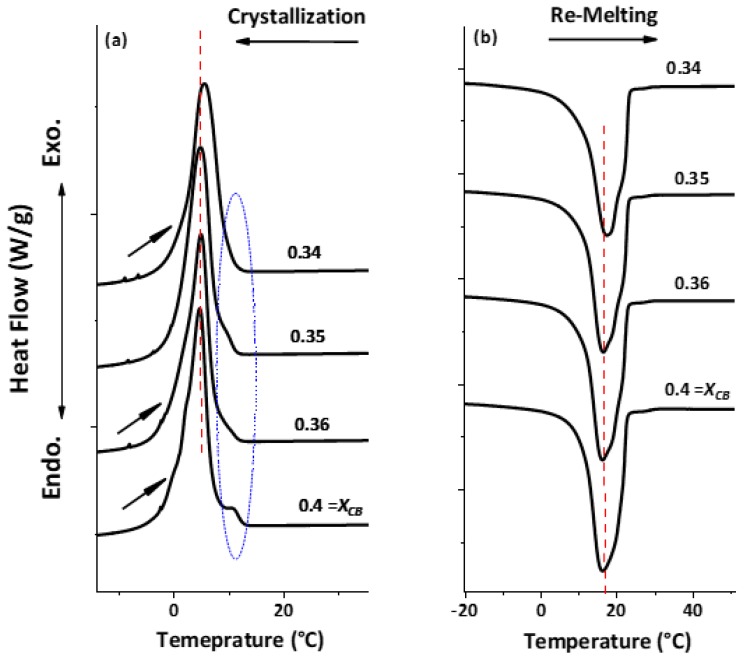
DSC analysis of CB/CO blends for the determination of eutectic mixture. The samples were prepared similarly like other blends and the same process was used for DSC measurements. (**a**) Dynamic crystallization from 90 °C to −50 °C. (**b**) Remelting of crystals formed in segment (**a**).

The crystallization and melting profiles of the above-defined mixtures are shown in Figure 5a,b. As the first melting process could not detect any thermogram because no crystallization occurred during 24 h, the first segment results are not shown here. The crystallization profile revealed that the shoulder peaks appeared on mixtures of X_CB_ = 0.40, 0.34 and 0.36 (highlighted with the arrow and blue ellipse), whereas for X_CB_ = 0.35 mixture, no specific shoulder peak was detected. However, in the case of the remelting segment, for the concentration of 0.40, 0.36 and 0.35 the peak sharpness did not show any specific change. Another observation in both crystallization and melting, was that the peak maxima were shifting towards a higher temperature as the concentration of CB was decreasing (shown by the dotted line). The eutectic mixture is when the mixture melts or solidifies homogeneously at one temperature, and this temperature is below the individual melting temperature. Therefore, our results of crystallization and melting together indicated that 0.35 of CB and 0.65 of CO represented a eutectic mixture, which melted at 16.7 °C. Unfortunately, we could not succeed in achieving a sharp peak in both the crystallization and melting process. The reason for such a behavior might be the mixture of different triacylglycerol in both fats, as pure Trilaurin (TL) from CO and POP from CB do not show any resemblance with the temperature profile of CB/CO blend (refer to supplementary Figure S3). This illustrates that the cause for eutectic mixtures of CB/CO blend was the different mixtures of triacylglycerol and not only individual TAG, which are present in a higher amount.

**Figure 6:**
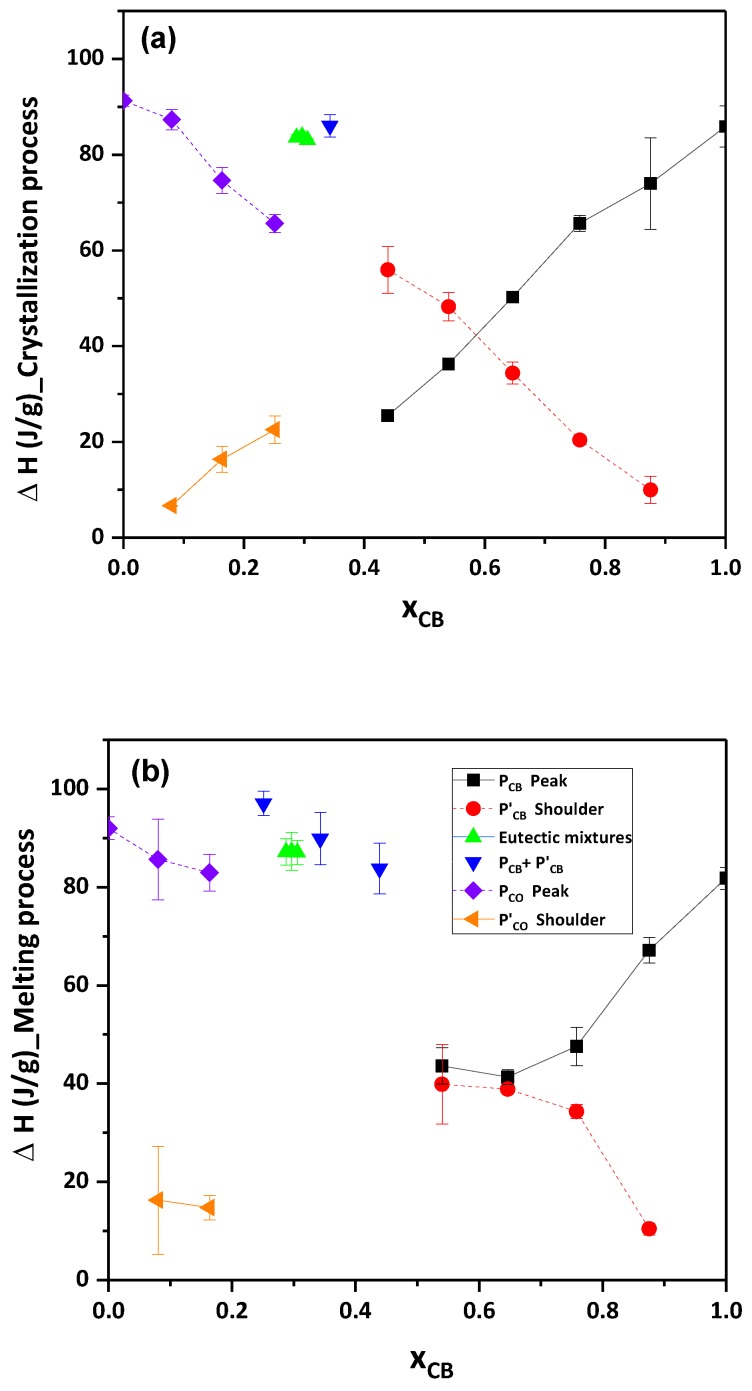
Enthalpy change of CB and CO during the crystallization (**a**) and remelting (**b**) process with respect to weight % of CB. black closed square = P_CB_, red closed circle = P’_CB_, green closed triangles = eutectic mixtures (X_CB_ = 0.36, 0.35, 0.34), blue closed reverse triangles = P_CB_+P’_CB,_ purple closed diamond = P_CO_, orange closed diamonds = P’_CO_.

Analysis of all thermograms was plotted in Figure 6a,b as enthalpy change with respect to X_CB_. By using STARe software, the enthalpy change was calculated for P_CB_, P’_CB_, P_CO_, P’_CO_ and one ∆H value for eutectic mixtures (refer to supplementary Figures S4 and S5). ∆H for P_CB_ decreased (linearly for crystallization and exponentially for melting) as the concentration of CB decreased in both processes, whereas, ∆H for P’_CB_ increased linearly and exponentially for crystallization and melting as the concentration of CB decreased in the mixture, respectively. Next, for mixture 0.40 in crystallization, only one ∆H value was calculated as the addition of P_CB_ and P’_CB_, similarly for melting process 0.50, 0.40 and 0.30 showed the broad peak, hence, one ∆H value was evaluated (noted as P_CB_ + P’_CB_). In both processes, ∆H for P_CO_ decreased as the concentration of CO decreased, however, P’_CO_ increased as the concentration was decreased in the mixture. From these results, we can conclude that P’_CB_ represented the CO phase in the blends, and P’_CO_ denoted the CB phase in the mixtures. Hence, from this hypothesis, one can say that there was phase separation in the case of CB and CO, even if both of them had a similar melting temperature. Only the eutectic mixture showed the homogeneity in two fats.

### 3.3. Pseudophase Diagram Comparison between CB/TS and CB/CO System

**Figure 7:**
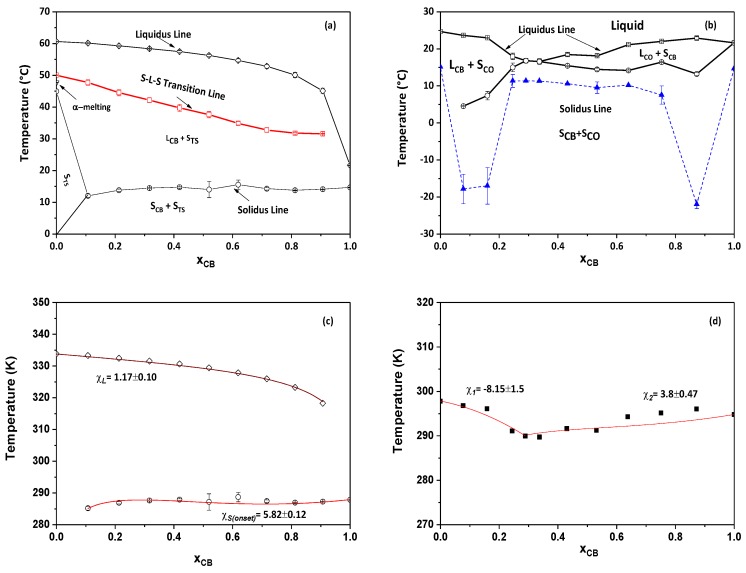
Comparison between pseudophase diagrams of (**a**) CB/TS blends (**b**) CB/CO blends. CB/TS phase diagram depicts a monotectic mixture of CB/TS, whereas CB/CO shows a eutectic mixture at X_CB_ = 0.35. (**c**,**d**) represents the fitting of phase diagram by using Bragg–Williams approximation for CB/TS and CB/CO blends, respectively.

The solid–liquid phase diagram for CB/TS and CB/CO was plotted by using DSC data from the remelting segment with respect to the mole fraction $\left(x_{CB}\right)$ in Figure 7a,b. In Figure 7a, the horizontal line (solidus line) achieved from onset point (P_CB_) and the monotonously increasing line (liquidus line) plotted from the melting profile of (P_TS_) described that the two fats were completely immiscible with each other. This solid-liquid phase diagram exhibits a monotectic behavior [41] where the solidus line occurs as a horizontal and the liquidus line increases from the melting temperature of pure CB to pure TS. The phase diagram shows that below the solidus line, solids of TS (S_TS_) and CB (S_CB_) coexist. However, at $x_{CB}=0$ the S_TS_ exists until α form melt. Above the solidus line, liquids of CB (L_CB_) and S_TS_ coexist until the S–L–S transition line. At this transition line, liquid transformed into solids of TS, and then the melting started. Hence, above this transition line, L_CB_, partial liquid of TS along with solids of TS coexisted. Above the liquidus line, both of the components were present in the liquid phase. A similar phase diagram was obtained in the case of POP/PPP mixtures [42]. According to their study, the phase diagram (POP/PPP) indicated that the interaction between monounsaturated (Sat–U–Sat) TAG and TAG of monosaturated fatty acid (PPP) could behave as a monotectic partial solid solution (as the difference in melting temperature of the TAG component is more). Therefore, our results agreed with their findings and it might be in the case of CB and TS that the mixture behaves as a monotectic partial solid solution.

Likewise, Figure 7b shows the solid–liquid phase diagram plotted for CB/CO blends from the DSC result. The liquidus line was constructed by considering peak temperature (P_CB_ and P_CO_ in Figure 4c and Figure 5b) in all blends, and the solidus line was achieved from onset temperature (blue triangles) and shoulder peak temperature (P’_CB_ and P’_CO_ in Figure 4c and Figure 5b). Up to the solidus line, both components were in solid phases and above the solidus line from composition X_CB_ = 0.35 to 1, liquid of CO (L_CO_) and solid of CB (S_CB_) coexisted, whereas, for composition range of X_CB_ = 0 to 0.35, liquid of CB (L_CB_) and solid-state of CO (S_CO_) coexisted. Above the liquidus line, both components were in the liquid phase.

The thermodynamic analysis of the liquidus and solidus line was performed by assuming nonideal mixing in both liquid and solid phases. Hence, Bragg–Williams approximation was considered instead of the Hildebrand model, which describes the ideal mixing behavior. In the present study of the CB/TS phase diagram, component A represents TS and component B is CB, whereas, in the CB/CO system, A denotes CB and B is CO. The fitting of Bragg–Williams approximation is shown in Figure 7c,d. Hence for fitting the liquidus line data for the CB/TS system, we used Equation (4) with the values of $∆H_{TS}\;$= 118,387.8 J/mol, $T_{TS}\;$*=* 333.76 K and *R* as 8.314 J/K.mol. Similarly for fitting the solidus line, we have considered Equation (5) with values of $∆H_{CB}\;$*=* 66,498.3 J/mol and $T_{CB\;}$*=* 294.785 *K*. For fitting the solidus line, we considered the onset temperature. After fitting the experimental data, χ*_L_* value was calculated as 1.17 + 0.10 kJ/mol (*R*^2^ >0.98) and χ*_s (onset)_* as 5.82 ± 0.12 kJ/mol (*R*^2^ >0.57) respectively. These high positive values indicated that the interaction energy of the CB-TS pair was more than the average of the CB–CB and TS–TS pair. This result suggests that some repulsive interaction acts between CB–TS, hence the energy needed is more. As a result, CB and TS are completely immiscible with each other in the solid phase. The possible reason for the higher interaction energy in CB–TS could be the structures of TAGs in CB and TS. Due to the presence of the oleic acid in the CB structure, the chain packing becomes complex in nature. Therefore, TS could have encountered difficulty in arranging themselves in a closely packed manner, whereas, in the case of mixing two TAGs with at least one unsaturated fatty acid (in both of them), there could be less difficulties to pack themselves closer [8]. Hence, in the case of CB/TS blends, CB could act as an impurity, and this might lead to a decrease in the temperature of TS as the concentration of CB increased in it. Similar results were also obtained by the study of oleic acid and stearic acid mixtures, where the stearic acid temperature decreased after the addition of oleic acid; however, there was no change in two polymorphs (*α* and *γ*) of oleic acid’s temperature profile [25].

For thermodynamic analysis of the CB/CO phase diagram, Equations (4) and (5) were used from the composition range of $x_{E}\;\leq x_{CB}\leq 1$ and $0\;\leq x_{CB}\leq x_{E}$ respectively. Hence, the values of $∆H_{CB}\;=\;66,498.3\;J/\mathrm{mol},\;T_{CB}\;=\;294.785\;K,\;∆H_{CO}\;=\;57,217.78\;J/\mathrm{mol},\;T_{CO}\;=\;297.815\;K$ were considered from DSC results for fitting the experimental data. The fitting of the liquidus line is shown in Figure 7d. According to investigations by Abes et al., for binary mixtures of saturated fatty acids (SS/MM), the interaction parameter gave only one value for whole composition range [26]. However, in our studies, for composition range from 0 to 0.35 wt % of CB, the value of χ was −8.15 ± 1.15 kJ/mol (*R*^2^ >0.54) and for the other range was 3.8 ± 0.47 kJ/mol (*R*^2^ >0.59). Due to differences in molecular structure and its volume, this approximation is not very well suited for CB/CO blends. From these values, it can be described that for CO and mixture with CB, CB–CO pair is more compatible than CB–CB pair and CO–CO pair in the liquid phase. For CB/CO blends of composition range from X_CB_ = 0.35 to 1, the large positive value, lead to the hypothesis that CB–CO interaction energy is more and hence there is phase separation in the liquid phase; the reason for such behavior is their molecular structure. CB has monounsaturated TAGs, and because of oleic acid, the molecular length is reduced due to a kink of the double bond, and hence, the molecular length of CB and CO becomes similar to each other and thus leads to the CB–CO pair instead of like pairs. In the case of further composition from eutectic to 1, an arrangement of CO, having a mixture of short chained (C12, C10 and C8) saturated TAGs and CB as monounsaturated TAGs, could not pack densely due to presence of a high percentage of CB, which is not flexible in nature even, in a liquid state.

Although Bragg–Williams type approximations used in our study for understanding the phenomenological interaction between CB/TS and CB/CO blends and the results obtained were in agreement with DSC results, it is still unclear how these interactions specifically take place in such complex fat/oil mixtures. A precise molecular interpretation is difficult, since, in the original Bragg–Williams approach, the interaction parameters were defined by pseudopotentials between monomers only. In the mixtures of the fatty acids, most of the monomers are identical, i.e., carbon–carbon bond and esterified glycerol bond. The main difference of the energy mismatch has its origin in the appearing cis-double bonds and the different chain lengths of the fatty acids. Both cause a large number of “defects” in the different polymorphs, which cause a major contribution to the energy balance. Hence, the Bragg–Williams model can be considered, in general, only as a phenomenological model to describe the phase diagrams for fat blends.

Nevertheless, the fitting results lead to the prediction that such interactions could occur due to either aliphatic chain packing, of which several forms exist, and/or the difference in carbonyl group conformational changes [24]. Hence, precise pinpointing of the interaction causing such behavior in both of our systems is challenging. Additionally, according to our data, we can say that this approximation does not take into consideration the various polymorphic states that exist in several other systems used in such studies, and hence makes it rather difficult to comprehend the interactions between distinct fat/oil blends.

### 3.4. Morphological Studies of CB/TS and CB/CO

**Figure 8:**
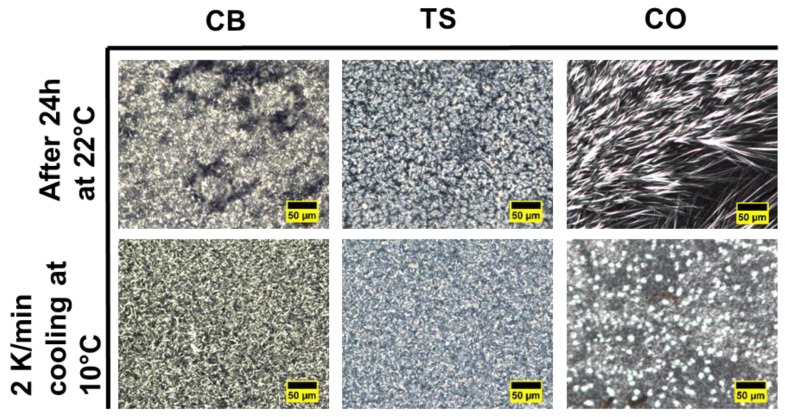
PLM analysis of pure fats after isothermal crystallization at 22 °C for 24 h and dynamic crystallization at 10 °C by cooling down with 2 K/minute rate. Scale bar = 50 µm.

The crystal morphology of CB/TS and CB/CO blends has been captured by PLM. Figure 8 shows the crystal morphology of pure CB, CO and TS after 24 h at 22 °C and after heating this sample to 90 °C and cooling down to 10 °C with 2 K/minute rate.

The pictures captured after 24 h at 22 °C, show the crystal formation after the isothermal crystallization process; hence, one can understand the morphology of the crystals, which were melted during the DSC melting segment (Figure 2a and Figure 4a). According to DSC results, for pure CB, the mixture of form IV (β_1_′) and form V (β_2_) was formed after 24 h at 22 °C, and the visualization of these polymorphs can be viewed in Figure 8. The morphology can be described as a high number of small size crystallites along with some spherulites having a needle-like structure at the periphery. In the case of pure TS, a spherulitic crystal network was formed. For pure CO, a sharp needle structure was observed at 22 °C; however, in the case of DSC, no sharp melting was observed.

After heating at 90 °C and cooling down to 10 °C, the formation of crystal morphology shows the visualization of the crystallization process from DSC (Figure 2b and Figure 4b). In Figure 8, for all pure components, the crystal size changed to smaller and denser as compared to isothermal crystallization. In the case of Pure CB, the crystals are the aggregation of small needles, and pure TS showed very small grainy structures. Similarly, for pure CO, the combination of small denser spheres and granular structure were formed.

**Figure 9:**
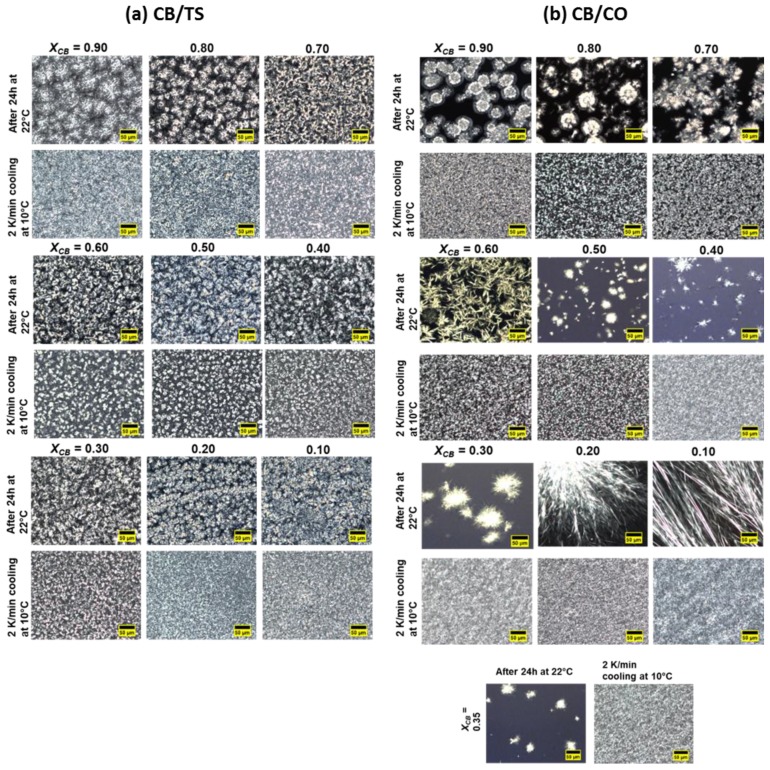
PLM analysis of (**a**) CB/TS blends, and (**b**) CB/CO blends after crystallizing isothermally at 22 °C for 24 h and then heating the sample to 90 °C and cooled down to 10 °C with 2 K/min rate for studying the effect of dynamic crystallization. Scale bar = 50 µm.

Furthermore, the effect of the addition of different fats on the crystal morphology of pure CB was further studied. Figure 9a shows the changes in crystal morphology after the addition of TS in CB for isothermal and dynamic crystallization. For X_CB_ of 0.90 and 0.80, at 22 °C after 24 h, spherulites consisting of needles were observed. For further compositions, the size of the spherulitic aggregates of needles was decreased as the concentration of TS increased. For dynamic crystallization, after the addition of TS in CB, crystal size reduced and formed the grainy structure for the composition of 0.90 and 0.80. From 0.70 to 0.30 of CB, the mixture of small spherulitic crystal structure and the grainy structure was observed. Again, for further compositions, smaller crystals were formed as the concentration of TS increased.

In contrast to CB/TS blends, a mixture of CB and CO showed various changes in crystal morphology depending on the CO concentration after 24 h. For X_CB_ = 0.90, spherulitic morphology was observed, likewise for 0.80 and 0.70 also showed the combination of a needlelike and spherulitic crystal nature. However, at 0.60, more sharp needles were viewed as compared to spherulitic. For 0.50 and 0.60 compositions, the size of spherulites was reduced, as was the amount of crystallinity of the whole sample blend. As the concentration of CO increased further, more sharp, needlelike crystal morphology was captured. After dynamic crystallization, the size of crystallites decreased drastically which is in agreement with the CB/TS results.

## 4. Summary

This study focused on the mixing behavior in CB/TS and CB/CO mixtures in different phases, on the basis of the solid–liquid phase diagram. DSC results depicted that CB/TS mixtures were completely immiscible in the solid phase and well mixed in a liquid state, whereas CB/CO showed eutectic mixture at 65 wt % of CO and 35 wt % of CB. Similarly, the thermodynamic analysis of the phase diagram resulted in: (1) the interaction for the mixed pair of CB/TS was energetically much more unfavorable than CB–CB and TS–TS pair; (2) in the case of CB/CO, the interaction of a mixed pair was favorable from 100 wt % CO to E_CB-CO_ (eutectic mixture) and unfavorable from E_CB-CO_ to 100 wt % CB.

Isothermal and dynamic crystallization processes altered the thermal properties and crystal morphology of CB/TS and CB/CO blends. From our results of the dynamic crystallization process, we can say that a decrease in melting temperature results in small granular crystal morphology of blends, whereas in isothermal crystallization, spherulitic to needlelike structures were formed that resulted in higher melting points. Thus we can conclude that the variations in thermal and morphological behavior of fat and oil (CB/TS and CB/CO) blends are highly dependent on: (1) crystallization kinetics, and (2) “defects” created in crystal structure arrangements due to monounsaturated part of CB.

To summarize, these results improve the understanding of molecular interactions in bulk fat–fat and fat–oil systems, as well as helping to explain the different thermal behaviors of various such mixtures. From an application point of view, this will ultimately help to engineer new types of fat-based products with desired properties, such as different organoleptic characteristics, which can be related to different mixing ratios.

## Figures and Tables

**Table 1:** Fatty acid composition of cocoa butter (CB) and coconut oil (CO).

Fatty Acid	Cocoa Butter (Weight %)	Coconut Oil (Weight %)
Caproic acid (C6:0)	-	0.72
Caprylic acid (C8:0)	-	7.75
Capric acid (C10:0)	-	5.64
Lauric acid (C12:0)	-	42.78
Myristic acid (C14:0)	0.09	17.64
Palmitic acid (C16:0)	25.32	9.61
Palmitoleic acid (C16:1ω7c)	0.24	-
Heptadecanoic acid (C17:0)	0.21	-
Stearic acid (C18:0)	36.74	2.87
Octadecenoic acid (C18:1-trans)	0.02	-
Oleic acid (C18:1ω9c)	32.48	9.23
Linoleic acid (C18:2ω6c)	2.88	2.99
Octadecadienoic acid(C18:2ω6-trans)	0.02	-
alpha Linolenic acid (C18:3ω3c)	0.17	0.3
Arachidic acid (C20:0)	1.09	0.1
cis-11-Eicosenoic acid (C20:1ω9c)	-	0.09
Behenic acid (C22:0)	0.18	-
Lignoceric acid (C24:0)	0.1	-
Saturated fatty acids	63.73	87.11
Monounsaturated fatty acids	33.07	9.53
Polyunsaturated fatty acids	3.05	3.29
Trans fatty acids	0.04	<0.01
omega-3 fatty acids	0.17	0.3
omega-6 fatty acids	2.88	2.99

**Table 2:** TAG composition of CB.

TAGs in CB	Conc. (wt %)	TAGs in CB	Conc. (wt %)
PPP	0.1	PLL	0.4
MOP	0.1	SSS	0.3
PPS	0.5	SOS	26.5
POP	16.7	OOS	2.2
PLP	1.8	OOO	0.2
PSS	0.6	SLO	0.3
POS	39.8	OLO	0.1
POO	1.7	SLL	0.2
PLS	3.4	LLO	<0.1
PLO	0.3	LLL	<0.1

P = palmitic acid; M = myristic acid; O = oleic acid; S = stearic acid; L = linoleic acid.

## Supplementary Materials

The following are available online at https://www.mdpi.com/2304-8158/9/3/327/s1, Figure S1: Area under the peaks for CB (50%)+TS (50%) is shown, Section 2: Supplementary reasoning for Section 3.2.1, Figure S2: SFC of CB/CO blends at 22°C after 24 h showed eutectic behavior, Figure S3: The DSC thermogram for pure components of CB (POP) and CO (Trilaurin) showed no eutectic behavior, Figure S4: Area under the curves for P_CO_ and P’_CO_ for CB (10%)+CO (90%), Figure S5: Area under the curves for P_CB_ and P’_CB_ for CB (90%) + CO (10%).
