# Supplementary material for: Comparative Study on Mixing Behavior of Binary Mixtures of Cocoa Butter/Tristearin (CB/TS) and Cocoa Butter/Coconut Oil (CB/CO)
Source: Foods. 2020 Mar 11;9(3):327. doi: 10.3390/foods9030327 (PMC7142511; doi:10.3390/foods9030327)

1) **Supplementary Figure S1-** CB/TS (Section 3.2.1) – In case of blends of CB/TS, the enthalpy change was calculated by considering the area under the peaks (P_{CB}), (P_{TS}) for CB and TS respectively. This figure represents the 50/50 *w/w* % of CB/TS and the area under the curves is calculated by using STARe software from Mettler Toledo DSC device. For crystallization process, two individual peaks were selected as P_{CB} and P_{TS}. For re-melting process, two endothermic for P_{CB} and P_{TS} and one exothermic for solid-liquid-solid transition was considered. This is the supplementary data for Figure 2 from main manuscript. The similar process was used for estimation of rest of the blends for estimation of enthalpy change during crystallization and melting processes.

Figure S1: Area under the peaks for CB (50%)+TS (50%) is shown

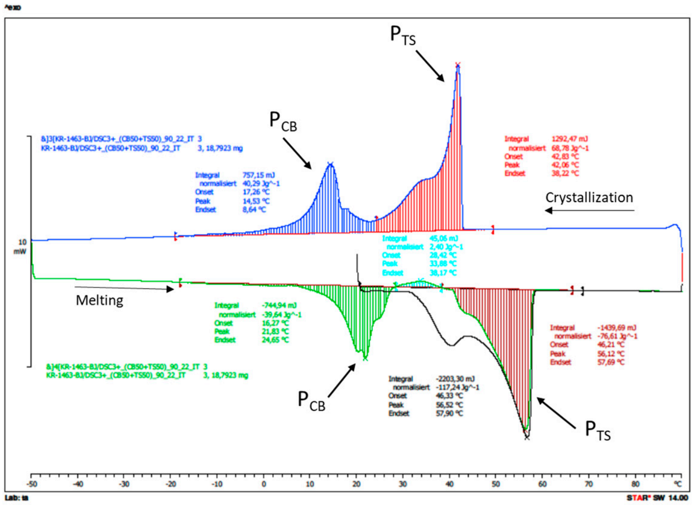

2) **Supplementary reasoning for section 3.2.1-** Why the change in enthalpy for S-L-S transition / melt mediated transition was almost constant in all blends (section 3.2.1)

To explain this behavior, the possible hypothesis could be that there might be always similar amount of one crystal form (for instance Form I) is forming while crystallizing process and as it's a non-equilibrium process, it tries to go towards the stable form while temperature increase and hence same energy is required in all blends for recrystallization. However, we still need to do further investigations to prove this hypothesis.

3) **Supplementary Figure S2:** Solid fat content of CB/CO blends after 24 h at 22°C was measure by using solid state high frequency NMR, which are in agreement with the DSC results (section 3.2.2).

Figure S2: SFC of CB/CO blends at 22°C after 24 h showed eutectic behavior

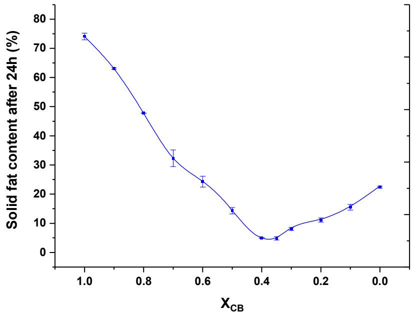

4) **Supplementary Figure S3-** Two components of having higher concentration in CB and CO were analysed by using DSC in order to find out the mixing behaviour of them. POP (CB) and Trilaurin (CO) were mixed in different ratios and perform DSC. However, their mixing behaviour showed no eutectic as CB and CO mixtures (section 3.2.2). The experiment was performed for 4 different blends of POP and Trilaurin (TL), are as- POP (60%) + TL (40%), POP (50%) + TL (50%), POP (40%) + TL (60%), POP (35%) + TL (65%).

Figure S3: The DSC thermogram for pure components of CB (POP) and CO (Trilaurin) showed no eutectic behavior.

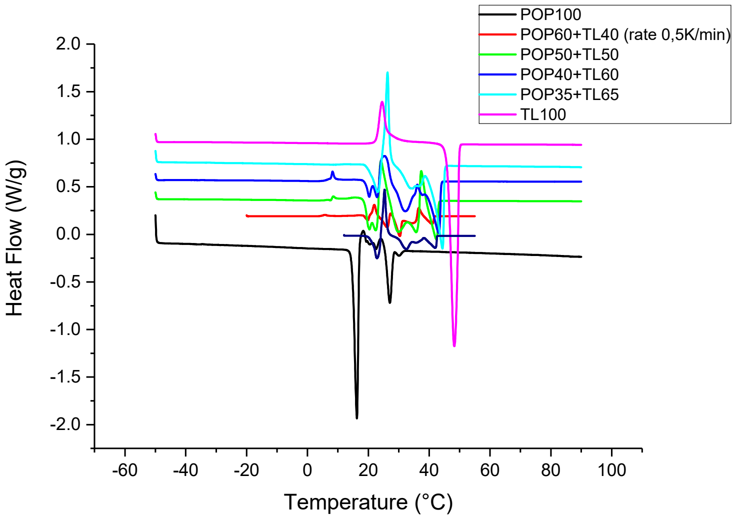

5) In case of blends of CB/CO, the enthalpy change was calculated by considering the area under the peaks (P_{CO}) and (P'_{CO}) and (P'_{CB}) for CB, CO, shoulder peaks of CO and shoulder peaks of CB respectively. In the below given figures the examples for two blends are shown.

a) **Supplementary Figure S4-** CB/CO (section 3.2.2) – This figure represents the 10/90 *w/w* % of CB/CO and the area under the curves are calculated by using STARe software from Mettler Toledo DSC device. For crystallization and re-melting processes, one peak as P_{CO} and shoulder peak P'_{CO} for CB fraction was considered. This is the supplementary data for Figure 4 from main manuscript.

Figure S4: Area under the curves for P_{CO} and P'_{CO} for CB (10%)+CO (90%)

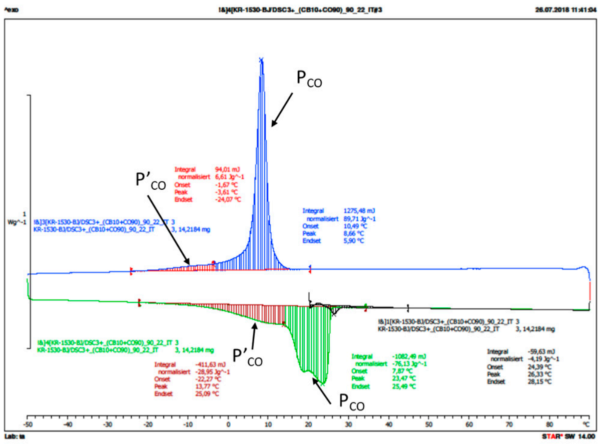

b) **Supplementary Figure S5-** CB/CO (section 3.2.2) – This figure represents the 90/10 *w/w* % of CB/CO and the area under the curves is calculated by using STARe software from Mettler Toledo DSC device. For crystallization and re-melting processes, one peak as P_{CB} and shoulder peak P'_{CB} for CO fraction was considered. This is the supplementary data for Figure 4 from main manuscript. The similar process was used for estimation of rest of the blends for estimation of enthalpy change during crystallization and melting processes.

Figure S5: Area under the curves for P_{CB} and P'_{CB} for CB (90%) + CO (10%)

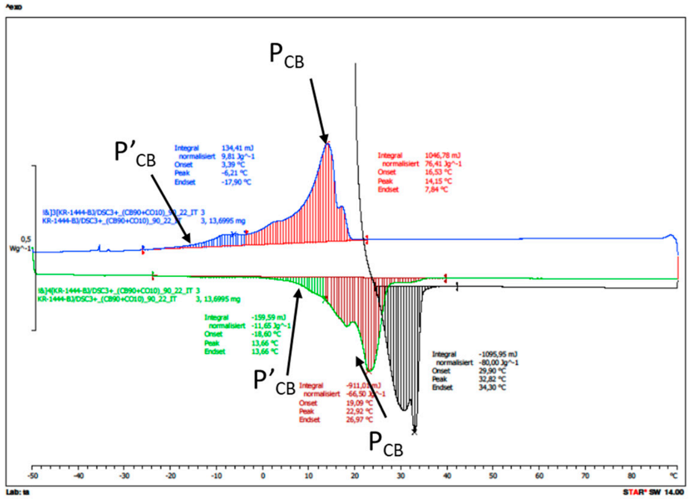

Supplement: Supplementary file 1 [file foods-09-00327-s001.pdf]
